# Investigating *PRPH2*-associated retinal degeneration patient-reported outcomes in vision-related quality of life

**DOI:** 10.1186/s12886-026-05011-4

**Published:** 2026-06-18

**Authors:** Gillian A. Folk, Naomi E. Wagner, Evan H. Walker, Shyamanga Borooah

**Affiliations:** 1 School of Medicine, University of California San Diego, La Jolla, CA USA; 2 Jacobs Retina Center, Shiley Eye Institute, University of California San Diego, 9415 Campus Point Drive, La Jolla, CA 92093-0946 USA; 3 Viterbi Family Department of Ophthalmology and Shiley Eye Institute, University of California San Diego, La Jolla, CA USA

**Keywords:** Quality of life, VFQ-25, Patient-reported outcome measures, *PRPH2*-associated retinal degeneration, Inherited retinal disease

## Abstract

**Background:**

*PRPH2-*associated retinal degeneration (PARD) is one of the most common causes of inherited retinal disease and can impair visual function and quality of life. While genetic and clinical features of PARD have been described, patient-reported experiences of visual symptoms remain less well characterized. This study aims to evaluate self-reported visual function and quality of life among individuals with genetically confirmed PARD.

**Methods:**

This cross-sectional study surveyed patients with confirmed PARD at a single academic center using the National Eye Institute Visual Function Questionnaire-25 (VFQ-25). The questionnaire was administered digitally and assessed 11 vision-related domains, including general vision, near and distance activities, and vision-related mental health. A composite score was calculated as the mean of all subscale scores excluding the general health item. Descriptive statistics were used to summarize responses, with higher scores indicating better perceived visual function.

**Results:**

Twenty-two participants completed the VFQ-25. The median (IQR) composite score was 76.9 (16.4), reflecting moderate perceived visual dysfunction. The highest-rated domains were ocular pain (87.5 (37.5)) and color vision (100.0 (0.0)). Domains related to functional activities demonstrated greater impairment, including general vision (80.0 (20.0)), near activities (79.2 (39.6)), distance activities (75.0 (29.2)), and driving (75.0 (33.3)). The vision-specific mental health domain had the lowest score (59.4 (40.6)), underscoring the psychological impact of PARD.

**Conclusions:**

Individuals with PARD report significant challenges in visual function and emotional well-being. These results highlight the importance of incorporating patient-reported outcome measures into the clinical characterization of PARD and the design of future therapeutic studies.

**Supplementary Information:**

The online version contains supplementary material available at https://doi.org/10.1186/s12886-026-05011-4.

## Introduction

The gene peripherin-2 *(PRPH2)* is located on chromosome 6 and encodes the protein peripherin-2, which localizes to the rim regions of rod and cone disc outer segments and lamellae [1–3]. PARD is among the most common forms of inherited retinal disease (IRD), with pathogenic variants identified in approximately 3%-5% of IRD cases [4–6]. It is estimated to affect between 6,000 and 22,000 individuals in the United States alone and up to 200,000 people worldwide [6]. Over 300 pathogenic or likely pathogenic variants have been identified, which are typically inherited in an autosomal dominant pattern [2, 7]. PARD can result in significant visual impairment degeneration [3, 8, 9]. *PRPH2* pathogenic variants result in diverse clinical presentations, including pattern dystrophy, macular dystrophy, central areolar choroidal dystrophy (CACD), pseudo-Stargardt disease, cone dystrophy, cone-rod dystrophy, and retinitis pigmentosa [2, 10–12]. The age of onset for autosomal dominant PARD is typically in adulthood, in the fourth or fifth decade, though this can also have great variability [4, 12]. Less commonly, autosomal recessive *PRPH2-*related disease is reported in individuals homozygous or compound heterozygous for pathogenic *PRPH2* variants, and more severe, early-onset phenotypes such as Leber congenital amaurosis (LCA) have been observed in these cases [12].

Currently, there are no FDA-approved treatments for PARD. However, there are several therapeutic strategies, including gene augmentation, gene editing, and RNA-based therapies, that are currently under investigation [6, 9, 13]. Currently, there is only one clinical trial aimed at targeting a specific *PRPH2* mutation [NCT: 07177196]. Understanding the unmet needs of individuals with PARD, and how the condition impacts their daily functioning and quality of life, is important for guiding future treatment directions and to holistically manage patients.

Although vision in patients with PARD is routinely assessed in the clinic, standard measures such as best corrected visual acuity, optical coherence tomography, fundus imaging and visual field testing do not fully capture subjective aspects of vision-related changes, including quality of life and daily functioning [14]. To address this gap, the National Eye Institute Visual Function Questionnaire (NEI VFQ-25) was used in this study to evaluate patient-reported visual outcomes (Supplementary Document 1). This survey was developed to monitor chronic eye conditions such as age-related cataracts, age-related macular degeneration, diabetic retinopathy, primary open-angle glaucoma, cytomegalovirus retinitis, or low vision from any cause [15, 16]. However, the NEI VFQ-25 has previously demonstrated effective evaluation in broader retinal disease cohorts and low vision, supporting its potential utility in further understanding its utility in PARD [15, 16].

The aim of this study is to characterize the impact of PARD on visual function and quality of life from the patient’s perspective. To our knowledge, the VFQ-25 has not yet been applied specifically to this patient population. Therefore, this study provides novel patient-reported outcomes that may complement clinical findings and inform the design of future clinical trials and studies.

## Methods

### Patient cohort

The study included patients with a previously confirmed clinical and molecular diagnosis of PARD from a single academic center, identified from the inherited retinal dystrophy database. Demographic information, genetic testing results, and most recent best corrected visual acuity (BCVA) were collected for each patient. Those who did not respond to the survey were excluded from analysis, however their clinical, genetic and demographic characteristics noted.

### Study design

This was a non-interventional, cross-sectional clinical study conducted at a single academic center. Patients were invited to complete an anonymous web-based survey between July and August 2025. A digital format was chosen to maximize accessibility and convenience, with specific design features (large text and side-by-side format) implemented to accommodate individuals with low vision. The survey was administered via a secure Qualtrics link and included the NEI VFQ-25 questions.

### Ethics approval and consent to participate

The study was conducted in accordance with the Declaration of Helsinki. All study participants were recruited from an IRB-approved longitudinal studies (#805734 and #171460), and all agreed to complete the survey after being briefed on the study’s purpose and procedures.

### NEI VFQ-25

The NEI VFQ-25 is a validated survey developed by RAND that contains 25 questions that assess 11 vision-related subscales: general vision, near vision, distance vision, driving, peripheral vision, color vision, ocular pain, vision-specific role difficulties, vision-specific dependency, vision-specific social functioning, and vision-specific mental health, along with a single general health question [15, 16].

Scores ranged from 0 to 100, with higher scores indicating better visual functioning. An overall composite score was calculated as the mean of the individual subscale scores, excluding the general health item. Across each domain, the distribution of scores was summarized using mean, standard deviation, median, minimum, and maximum values. All surveys were distributed in accordance with NEI stipulations and permission and scored by a single researcher.

### Statistical analysis

To visualize score variability, boxplots were generated for each domain, displaying the interquartile range, median, and overall spread of questionnaire responses. Boxplots were used to compare score distributions between domains via visual inspection. The statistical analysis was conducted using the R programming language for statistical computation, version 4.5.1 (R Foundation for Statistical Computing, Vienna, Austria).

## Results

### Patient demographics & characteristics

A total of 22 identified patients with PARD were included in this cohort (Table 1). The mean age was 58.1 years (range, 31–78 years). Fourteen patients (63.6%) were female, and eight (36.4%) were male. The majority of patients self-identified as white (*n* = 15, 68.2%). Two patients (9.1%) identified as Hispanic, and one patient (4.5%) identified as Black/African American. One patient (4.5%) identified as Asian, and one (4.5%) as other. Ethnicity was recorded as unknown for two patients (9.1%). The best corrected visual acuity (logMAR) from the most recent visit, retinal disease phenotype, and pathogenic variant is summarized in Table 1. Color fundus images, fundus autofluorescence images, and optical coherence tomography of the macula for each subject are shown in Supplementary Fig. 1. Based on the WHO Distance Visual Impairment Categories (WHO), 3 patients experience severe distance vision loss or blindness in at least one eye. The mean logMAR score for the participants is 0.36 +/- 0.42.

**Table 1:** Patient demographics, most recent visual acuity, retinal disease phenotype, and pathogenic variants

Subject	Race	Ethnicity	Phenotype	Genetic Variant	Visual acuity (LogMAR)	Atrophy present?	Central involvement?
1	Other Race or Mixed Race	Hispanic	CACD Type 2	*PRPH2* c.514 C > T (p.Arg172Trp), heterozygous, pathogenic	0.00 OU	No	No
2	White	Not Hispanic	Multifocal PD	*PRPH2* c.246 C > A (p.Cys82*), heterozygous, pathogenic	0.30 OD; 0.40 OS	Yes	Yes, OS > OD
3	White	Not Hispanic	CACD Type 2	*PRPH2* c.623G > A (p.Gly208Asp), heterozygous, pathogenic	0.10 OD; 0.30 OS	No	No
4	White	Not Hispanic	RP with butterfly PD OS	*PRPH2* c.647 C > T (p.Pro216Leu), heterozygous, pathogenic	0.54 OD; 0.40 OS	No	Yes, CME OU, OD > OS
5	White	Not Hispanic	Multifocal PD	*PRPH2* c.828 + 3 A > T, heterozygous, pathogenic	1.30 OD; 0.50 OS	Yes	Yes
6	White	Not Hispanic	Multifocal PD	*PRPH2* c.629 C > G (p.Pro210Arg), heterozygous, pathogenic	0.30 OD; 0.00 OS	No	No
7	White	Not Hispanic	Multifocal PD	*PRPH2* c.469G > A (p.Asp157Asn), heterozygous, pathogenic	0.10 OU	Yes	No
8	White	Not Hispanic	CACD Type 2	*PRPH2* c.623G > A (p.Gly208Asp), heterozygous, pathogenic	0.50 OU	Yes	No
9	White	Not Hispanic	Multifocal PD	*PRPH2* c.629 C > G (p.Pro210Arg), heterozygous, pathogenic	0.00 OD; 1.10 OS	No	No
10	White	Not Hispanic	Multifocal PD	*PRPH2* c.422 A > G (p.Tyr141Cys), heterozygous, pathogenic	0.48 OD ; 1.9 OS	Yes	No
11	Black	Not Hispanic	Butterfly PD	*PRPH2* c.136 C > T (p.Arg46*), heterozygous, pathogenic	0.30 OD; 0.20 OS	No	No
12	Unknown	Unknown	CACD Type 4	*PRPH2* c.623G > A (p.Gly208Asp), heterozygous, pathogenic	0.20 OD; 0.30 OS	Yes	Yes
13	Unknown	Unknown	CACD Type 2	*PRPH2* c.424 C > T (p.Arg142Trp), heterozygous, pathogenic	0.00 OD; 0.10 OS	No	No
14	White	Not Hispanic	CACD Type 4	*PRPH2* c.623G > A (p.Gly208Asp), heterozygous, pathogenic	0.20 OD; 0.90 OS	Yes	Yes
15	White	Not Hispanic	Multifocal PD	*PRPH2* c.422 A > G (p.Tyr141Cys), heterozygous, pathogenic	0.00 OU	No	No
16	White	Not Hispanic	Multifocal PD	*PRPH2* c.629 C > T (p.Pro210Leu), heterozygous, pathogenic	0.20 OU	Yes	No
17	Asian	Not Hispanic	CACD Type 4 OD; CACD Type 2 OS	*PRPH2* c.424 C > T (p.Arg142Trp), heterozygous, pathogenic	1.18 OD; 1.40 OS	Yes	Yes
18	Other Race or Mixed Race	Not Hispanic	Butterfly PD	*PRPH2* c.948del (p.Trp316*), heterozygous, pathogenic	0.00 OD; 0.10 OS	No	No
19	White	Not Hispanic	CACD Type 4	*PRPH2* c.623G > A (p.Gly208Asp), heterozygous, pathogenic	0.48 OD; 0.18 OS	Yes	Yes
20	White	Not Hispanic	Multifocal PD OD; Butterfly PD OS	*PRPH2* c.702 C > A, p.(Tyr234*), heterozygous, likely pathogenic	0.30 OD; 0.50 OS	No	No
21	Other Race or Mixed Race	Hispanic	CACD Type 2 OD; CACD Type 3 OS	*PRPH2* c.424 C > T (p.Arg142Trp), heterozygous, pathogenic	0.00 OU	Yes	Yes
22	White	Not Hispanic	Multifocal PD	*PRPH2* c.303 C > A (p.Tyr101*), heterozygous, likely pathogenic	0.40 OD; 0.00 OS	Yes	Yes

Note: participant 6 is the child of participant 9 and participant 14 and 19 are siblings

Among the participants, there was a wide spectrum of PRPH2-associated retinal phenotypes. The most common phenotype was multifocal pattern dystrophy (*n* = 9, 40.9%), followed by central areolar choroidal dystrophy (CACD) type 2 (*n* = 4, 18.2%) and CACD type 4 (*n* = 3, 13.6%). Additionally, 3 patients (13.6%) demonstrated inter-eye phenotypic variability, with differing phenotypes between eyes. This distribution highlights the clinical heterogeneity of PARD in our cohort (Table 1).

### Composite score

**Fig. 1:**
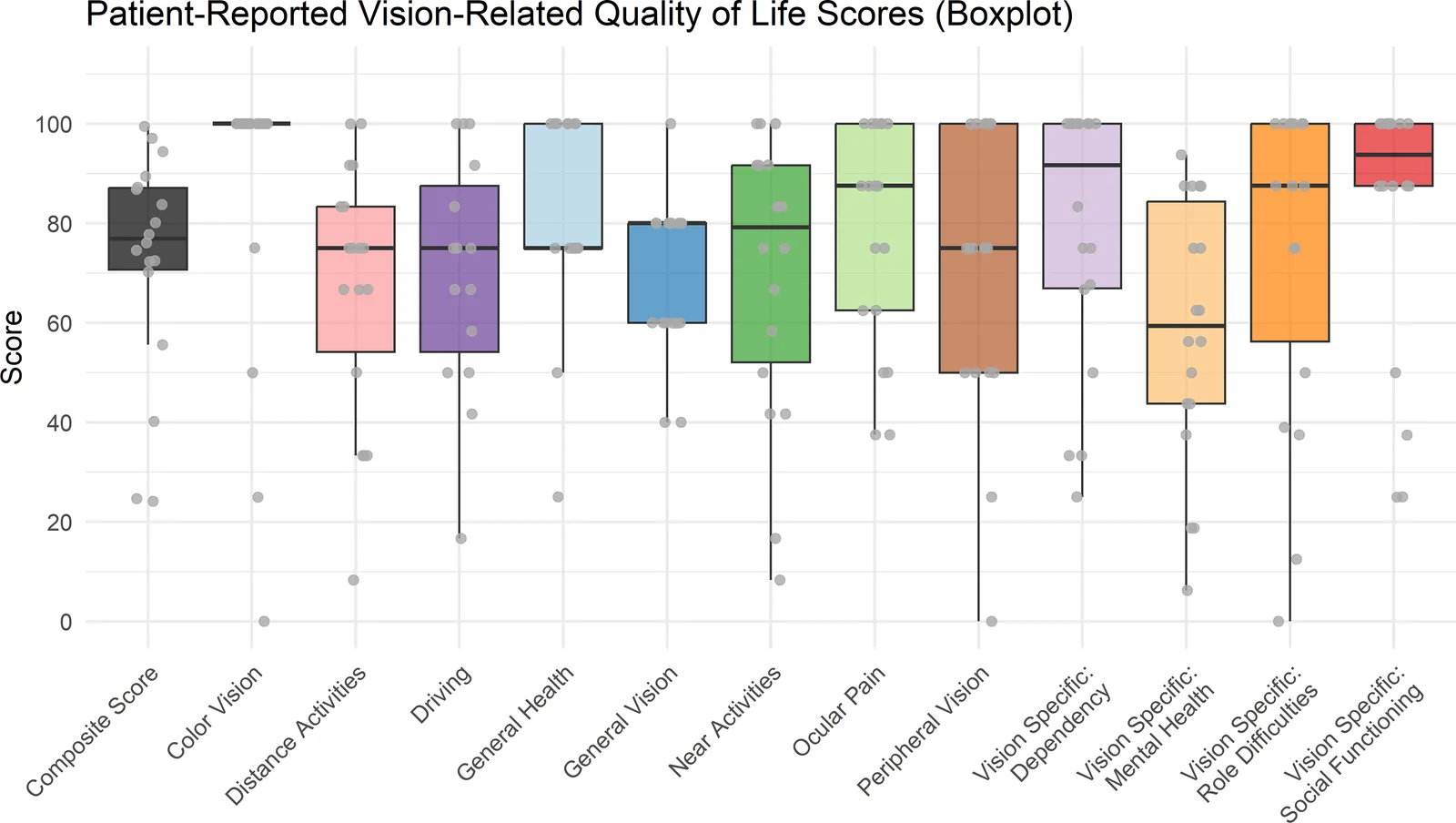
Distribution of VFQ-25 domain scores displayed as boxplots, where boxes represent the interquartile range (IQR) with the median indicated by the center line, and individual observations overlaid as points, illustrating the heterogeneity of patient-reported visual function across domains

A total of 22 patients completed the NEI VFQ-25 questionnaire. The average subscale and composite scores from the VFQ-25 demonstrated the impact of PARD on vision-specific quality of life across different domains (Table 2; Fig. 1). The median (IQR) composite score was 76.9 (16.4), indicating a moderate degree of self-reported visual functioning.

**Table 2:** National eye institute visual function questionnaire–25 (VFQ-25) domain scores. Reported values include mean, standard deviation (SD), median, minimum, and maximum scores for each domain, as well as the composite score

Category	Number of Items in Domain	Mean Score	SD	Median	IQR	Minimum	Maximum
Composite Score		72.6	22.6	76.9	16.4	24.1	99.4
Color Vision	1	86.1	30.0	100.0	0.0	0.0	100.0
Distance Activities	3	67.1	25.8	75.0	29.2	8.3	100.0
Driving	3	70.0	23.9	75.0	33.3	16.7	100.0
General Health	1	80.6	20.2	75.0	25.0	25.0	100.0
General Vision	1	70.0	15.7	80.0	20.0	40.0	100.0
Near Activities	3	69.9	28.2	79.2	39.6	8.3	100.0
Ocular Pain	2	77.8	22.5	87.5	37.5	37.5	100.0
Peripheral Vision	1	70.8	28.8	75.0	50.0	0.0	100.0
Vision Specific:							
Dependency	3	78.3	26.8	91.7	33.1	25.0	100.0
Mental Health	4	58.3	26.6	59.4	40.6	6.2	93.8
Role Difficulties	2	73.7	32.2	87.5	43.8	0.0	100.0
Social Functioning	2	81.9	27.2	93.8	12.5	25.0	100.0

### Subscale scores

The median (IQR) score for general health was 75.0 (25.0), and 80.0 (20.0) for general vision. Ocular pain yielded a median score of 87.5 (37.5), while color vision was the highest-rated domain at 100.0 (0.0), suggesting that basic visual functions were relatively preserved.

Domains related to functional visual tasks demonstrated greater impact. Median scores for near activities and distance activities were 79.2 (39.6) and 75.0 (29.2), respectively. Driving resulted in a median score of 75.0 (33.3), and peripheral vision was rated at 75.0 (50.0), reflecting moderate limitations in spatial orientation and mobility.

Among vision-specific subscales, mental health had the lowest median score (59.4 [IQR = 40.6]), underscoring the psychological burden of visual impairment. Role difficulties yielded a median of 87.5 (43.8), while dependency and social functioning were less affected, with median scores of 91.7 (33.1) and 93.8 (12.5), respectively.

## Discussion

To our knowledge, this study is the first to specifically apply the NEI VFQ-25 to a cohort of patients with PARD. Our results demonstrate that individuals with PARD report a moderate degree of functional vision impairment, with a median composite VFQ-25 score of 76.9. Subscale analysis revealed that patients perceived the greatest difficulties with near and distance activities, driving, and peripheral vision. The lowest median score was observed in the vision-specific mental health domain, highlighting the psychological burden associated with PARD. In our cohort, patients demonstrated wide variability in VFQ-25 scores. This variability likely reflects the wide spectrum of phenotypes in PARD, including differences in disease severity, progression, and visual function.

These findings provide new insight into the challenges that patients with PARD may encounter, complementing prior clinical descriptions of disease phenotype and progression. In addition, our results add evidence that patient-reported outcomes capture aspects of ophthalmic diseases not fully reflected by more traditional clinical measures like visual acuity or optical coherence tomography.

When compared to previous literature on VFQ-25 scores in age-related macular degeneration, the patient-reported burden of PARD appears substantial. Composite, social functioning, role limitations, dependency, and driving scores in our cohort were similar to those reported for advanced AMD [17]. However, PARD patients reported notably lower color vision (86.1 vs. 86.5–99.2), peripheral vision (70.8 vs. 84.7–96.1), mental health (58.3 vs. 59.6–80.2), and ocular pain (77.8 vs. 87.5–95.3) scores than those reported for advanced AMD patients with ranges representing both unilateral and bilateral presentations [17]. These findings suggest that the functional impact of PARD is comparable to or exceeds that of advanced AMD, demonstrating the severity of this disease from the patient perspective. The relatively high functional burden observed in PARD compared with advanced AMD may be explained by differences in disease topography and structural involvement. PRPH2-associated disease often leads to widespread disruption of photoreceptor outer segment architecture affecting both macular and peripheral retina, whereas AMD is primarily macula-centered with relative peripheral sparing until late stages. In addition, the patchy and heterogeneous nature of outer retinal degeneration in PARD may result in disproportionate impairment of contrast sensitivity, scotopic vision, and spatial integration, which are not fully captured by visual acuity measures but substantially impact patient-reported visual function.

To our knowledge, only one similar study, Fischer et al. (2023), included subjects with PARD (*n* = 2) to assess the Visual Symptom and Impact Outcomes patient-reported outcome (ViSIO-PRO) and observer-reported outcome (ViSIO-ObsRO) assessment, which measure health-related quality of life [18]. Responses were not specified for PARD subjects.

Because previously published studies report cohort scores as means rather than medians, we compared our cohort’s mean scores with those reported in the existing literature. Comparing patient-reported outcomes in PARD in prior IRD literature, patients with retinitis pigmentosa (RP) have been reported to have lower VFQ-25 scores than those observed in our PARD cohort. Watanabe et al. (2022) reported a mean composite VFQ-25 score of 42.0 in RP, compared with 72.6 in our cohort [19]. Similarly, Altinbay et al. (2021) found lower VFQ-25 domain scores across multiple subscales in RP relative to our results, including general vision (36.1 vs. 70.0) [14]. These differences likely reflect distinctions in disease pathogenesis and clinical course, as RP is typically characterized by progressive rod-cone degeneration with early and prominent peripheral visual field loss that ultimately progresses to central involvement, whereas PARD encompasses a broader and more heterogeneous spectrum of phenotypes with variable macular and peripheral involvement and rates of progression.

When compared with *CRB1*-associated retinal dystrophy, VFQ-39 scores (an expanded version of the VFQ-25) were also generally lower than those observed in our PARD cohort across most domains [20]. The only exception was ocular pain, where scores were slightly lower in PARD (77.8 vs. 80.5). It should be noted that differences in questionnaire versions (VFQ-25 vs. VFQ-39) and underlying disease severity may also limit direct comparability across cohorts.

Collectively, these findings suggest that while PARD is associated with substantial functional impairment, the overall patient-reported burden in this cohort appears less severe than that reported in more progressive or uniformly degenerative IRDs such as typical RP or CRB1-associated retinal dystrophy. However, given differences in study design, disease heterogeneity, and outcome instruments, these comparisons should be interpreted cautiously.

A key result of this study was that patients with PARD reported particularly low scores in the mental health domain (median of 59.4), indicating the notable emotional burden associated with this disease. These findings emphasize that the impact of PARD affects emotional well-being, independence, and daily life activities. Therefore, the mental toll of living with PARD should be an important consideration for both clinical care and future therapeutic trials, especially given the current lack of therapeutic options. However, a possible confounding factor to this finding is that individuals in this cohort have diagnosed depression, possibly unrelated to PARD. Pre-existing mental health conditions may have been associated with this increase in reported mental health symptoms. This score suggests that clinicians should include comprehensive strategies during visits that address not only visual impairment but also quality of life and psychological support.

The observed ocular pain scores in this cohort were somewhat unexpected, given that PARD is not typically associated with pain as a primary feature. However, the ocular pain domain of the NEI VFQ-25 captures symptoms such as ocular discomfort, dryness, and irritation, which may be influenced by factors independent of retinal pathology. Coexisting ocular surface disease, environmental factors, or age-related changes may contribute to these symptoms. Additionally, variability in patient perception and reporting of discomfort may further explain this finding. Therefore, the lower ocular pain scores observed in this cohort may not directly reflect retinal disease severity but rather concurrent ocular or systemic factors.

As gene-based therapies and other novel interventions emerge [9, 13], test measures that reflect patient reported outcomes are essential. Structural endpoints and visual acuity alone may underestimate functional and emotional burden. Patient-reported outcome measures are increasingly emphasized in ophthalmic drug development, with regulatory guidance from the U.S. Food and Drug Administration supporting their use as clinically meaningful endpoints that capture treatment benefit from the patient perspective [21]. Our findings suggest that future clinical trials should consider functional burden when defining inclusion criteria, assessing disease severity, and evaluating treatment effects. This data may also guide subgroup stratification and monitoring of treatment efficacy in PARD.

This study has several limitations. First, the small sample size, expected for rare conditions such as genetically confirmed PARD, limits the statistical power of the study. Although 29 patients were identified in the database, only 22 responded to the questionnaire, further reducing the analyzable cohort. This selection bias may have influenced the findings, as participation was voluntary and only a subset of eligible patients completed the survey. Differences in disease severity between responders and non-responders may have affected the results of this current study. Second, recruitment was limited to a single academic care center, which may limit generalizability. Another limitation is the cross-sectional design, which captures patient experiences at only a single time point. Additionally, because the questionnaire was administered anonymously, we were unable to correlate patient-reported outcomes with phenotypic subtype. A further limitation of this study is the use of a general vision-related quality-of-life instrument (NEI VFQ-25) rather than an inherited retinal disease-specific questionnaire, such as the Michigan Retinal Degeneration Questionnaire (MRDQ). While the VFQ-25 is widely validated and enables comparison across heterogeneous ocular diseases, it may be less sensitive to certain disease-specific functional domains relevant to inherited retinal dystrophies.

Future research should prioritize multicenter collaboration to evaluate larger cohorts of patients with PARD subtypes. Also, IRD-specific patient-reported outcome measures, such as the MRQ, may provide additional sensitivity to disease-specific functional changes. In addition, longitudinal designs will allow for assessment of changes in quality of life over time and enhance phenotype correlations with patient reported outcomes.

## Conclusion

This study demonstrates that PARD substantially affects patient-reported quality of life, particularly in domains involving functional activities and emotional well-being. Patients reported experiencing significant limitations in daily activities and psychological burden. These findings emphasize the importance of incorporating patient-reported outcomes into both clinical care and future clinical trials. By emphasizing the perspectives of patients, the VFQ-25 provides a more comprehensive understanding of disease impact and can play a role in guiding future therapeutic strategies.

## Supplementary Information

Below is the link to the electronic supplementary material.


Supplementary Material 1: Supplemental Document 1: NEI VFQ-25 full questionnaire



Supplementary Material 2: Supplemental Figure 1: OU color fundus (OD Panel A, OS Panel B), fundus autofluorescence (OD Panel C, OS Panel D), and optical coherence tomography of the macula (OD Panel E, OS Panel F) for each subject. Subject order corresponds to that of Table 1


## Data Availability

The datasets used and/or analyzed during the current study are available from the corresponding author on reasonable request.

## Abbreviations

AMD: Age-Related Macular Degeneration
BCVA: Best Corrected Visual Acuity
IRD: Inherited Retinal Disease
IQR: Interquartile Range
logMAR: Logarithm of the Minimum Angle of Resolution
MRDQ: Michigan Retinal Degeneration Questionnaire
NEI: National Eye Institute
PARD: PRPH2-Associated Retinal Degeneration
PRPH2: Peripherin-2
RP: Retinitis Pigmentosa
VFQ-25: Visual Function Questionnaire-25
VFQ-39: Visual Function Questionnaire-39
ViSIO-ObsRO: Visual Symptom and Impact Outcomes Observer-Reported Outcome
ViSIO-PRO: Visual Symptom and Impact Outcomes Patient-Reported Outcome
